# Private Dental Schools in Iraq: A Real Threat to the Dental Profession

**Published:** 2020-01

**Authors:** Ammar N.H. ALBUJEER, Mohammad Reza KHAMI, Alya ALMAHAFDHA

**Affiliations:** 1 Nab’a Al-Hayat Foundation for Medical Sciences and Health Care, Najaf, Iraq; 2 School of Medicine and Dentistry, University of Santiago de Compostela, Santiago de Compostela, Spain; 3 School of Dentistry, Tehran University of Medical Sciences, Tehran, Iran; 4 Research Center for Caries Prevention, Dentistry Research Institute, Tehran University of Medical Sciences, Tehran, Iran; 5 School of Medicine, International Campus, Tehran University of Medical Sciences, Tehran, Iran

## Dear Editor-in-Chief

The mission of the majority of dental colleges around the world is to provide the high level of education and facilities to learn about the upstream factors that affect the health outcomes, such as personal behaviors, health care quality and access, social, cultural, economic factors, and to graduate dentists with the ability and desire to improve the health of all people by alleviating suffering and eliminating healthcare disparities through their leadership in patient care, research, education, health care administration, and the community (1, 2).

To achieve these missions appropriately, such authorities as to the ministry of health, ministry of higher education, accreditation bodies, etc. which are responsible for supervision and accreditation of dental colleges must have strict rules and regulations for establishment of the new colleges.

Unfortunately, in most developing countries such as Iraq, many conflicts of interest exist (3). These conflicts force the authorities to give license to establish dental colleges for financial profits without taking into account the international standards (4). Moreover, in Iraq, the new policy of Ministry of Higher Education in establishing dental colleges ignores the number of the dentist/population ratio according to international standard which is 1 dentist for 2,000 patient. The same trend occurred in Iran from 2005 to 2013, with an increase in the number of dental schools from 18 to 45. Although the trend then was controlled and stopped, still some problems related to rapid growth of the dental schools exist.

The tremendous increase in establishing new private colleges in the same province in Iraq threaten the quality of education mainly due to severe shortage in teaching staff. This, in turn, leads to graduation of unqualified dentists and low-quality oral health care services (5, 6). Thus, international dental profession associations have to take action in order to raise attention to this problem (7).
